# A Novel and Simpler Alkaline Hydrolysis Methodology for Extraction of Ferulic Acid from Brewer’s Spent Grain and its (Partial) Purification through Adsorption in a Synthetic Resin

**DOI:** 10.3390/foods9050600

**Published:** 2020-05-08

**Authors:** Pedro Ideia, Ivo Sousa-Ferreira, Paula C. Castilho

**Affiliations:** 1 CQM—Centro de Química da Madeira, Universidade da Madeira, Campus da Penteada, 9020-105 Funchal, Portugal; pedro.ideia@staff.uma.pt; 2 Centro de Estatística e Aplicações, Faculdade de Ciências, Universidade de Lisboa, 1749-016 Lisboa, Portugal; ivo.ferreira@staff.uma.pt

**Keywords:** ferulic acid, brewer’s spent grain, alkaline hydrolysis, adsorption, synthetic resin

## Abstract

This work aims to develop simpler methodologies of extracting ferulic acid (FA) from brewer’s spent grain (BSG). BSG is produced by brewing companies at high amounts all over the year and does not possess a direct application. Thus, its use as raw material for extraction of bioactive compounds has gained attention in the last years. FA has different interesting applications in cosmetics, food industry, and pharmaceutics. Several studies aim for its extraction from BSG by various methods, namely alkaline hydrolysis. In the present work, we suggest the use of autoclave to process higher amounts of BSG in a lab scale. A simplification of the regular post-hydrolysis procedures is also proposed to decrease the number of experimental steps and energy costs and to simultaneously increase the extraction yield (up to 470 mg of FA per 100 g of BSG). The adsorption of extracted FA in a synthetic resin is suggested as a partial purification method.

## 1. Introduction

Due to the great political and social pressure in reducing pollution arising from industrial activities, large companies no longer consider residues as a waste but as valuable raw materials for other processes [1].

Brewer’s spent grain (BSG) is the main solid by-product of brewing industry, produced during the wort elaboration step of beer production [1]. BSG is produced in a ratio of 20 kg per 100 L of beer [2], and the worldwide production is around 38.6 × 10^6^ tons/year [3]. Despite being commonly used for animal and even human feed [4,5,6,7], excessive BSG availability is gaining attention for other applications, among which are the production or the extraction of high value added compounds, namely oligosaccharides [8], xylitol [9,10], and ferulic acid (FA) [11,12,13], a phenolic compound belonging to the hydroxycinnamic acids family.

Due to its physiological functions—anti-oxidant, anti-inflammatory, anti-thrombosis anti-microbial, and anti-cancer—as well as its protective effect against coronary disease, FA is considered one of the most important phenolic compounds [14]. Several applications are described, such as vanillin production [15], as preservatives [16,17], and as an ingredient for dermatologic lotions [14], among others. Because FA is covalently linked to the structure of lignocellulosic biomass by ether and ester bonds, conventional extraction techniques (e.g., solid–liquid extraction) are not effective in its separation from the matrix. Other techniques, such as alkaline hydrolysis, are needed in order to cleave these bonds and release FA. In fact, alkaline hydrolysis is able to cleave the lignin/phenolic-carbohydrate complexes structure, resulting in a phenolic portion, soluble sugars, insoluble lignin, and carbohydrates [18]. Other methods such as enzymatic hydrolysis were developed to recover ferulic acid from lignocellulosic biomass, namely wheat bran [19,20]. The main disadvantages of enzymatic hydrolysis are the cost of enzymes and/or the reaction time. Additionally, for the process to be efficient, control of reaction temperature and pH is required.

Different procedures for alkaline hydrolysis reactions are described in literature, namely those reported by Mussatto et al. (2007) [11] and McCarthy et al. (2013) [6]. The most common is to perform the reaction into auto-pressurized tubes or cylindrical stainless steel reactors at high temperatures and high pressures. Moreia and co-workers [13,21] suggested using microwave assisted extraction (MAE) to promote FA release from lignocellulosic materials, such as BSG. Despite the short time required for extraction, MAE’s main limitation is the small amount of BSG the system is able to process in each batch. The present work aimed to optimize FA extraction from BSG by the simplification of the methods described in literature, which resulted in reducing the time and the resources required for extraction. Additionally, extraction in an autoclave allows one to scale-up the FA extraction, bridging the limitations of other processes already described.

Currently, adsorption technology is widely used for the removal of organic compounds from aqueous solutions and heterogeneous mixtures. The main disadvantage associated with the most used adsorbents is the high regeneration cost. This has stimulated the research on new adsorbents such as macrobead synthetic resins, which may provide a cheap and effective chemical regeneration process [22].

Several authors refer to the adsorption of ferulic acid in resins such as Amberlite XAD-16 [23] or Lewatit-type resins [24], aiming at its purification. These last authors studied three different polystirene-based macroporous resins, Lewatit S6328 A (an anionic, strongly alkaline exchange resin), Lewatit S2328 (food grade cationic exchange, strongly acidic), and Lewatit S7968, a resin without functional groups, which gave the best performance on the adsorption of chlorogenic acids from artichoke residues with little sugar co-adsorption. A similar resin Lewatit VPOC1064 was chosen for purification of the extracted FA in the present work.

Our goal in this work was to use the principles of methods already reported and simplify the processes for cleaning as well as partially purify the FA extracted from BSG.

## 2. Materials and Methods

### 2.1. Chemicals and Reagents

All reagents and standards were of analytical reagent (AR) grade. Folin–Ciocalteu reagent, gallic acid (99%), and sodium hydroxide (98%) were purchased from Panreac (Madrid, Spain). Ferulic acid (99%) and sodium carbonate (99.8%) were from Sigma-Aldrich (St. Louis, MO, USA). Absolute ethanol was obtained from Riedel-de Haën, acetonitrile (99.9%) from Fisher Scientific, and formic acid (≥98%) from Sigma-Aldrich. Synthetic resin (Lewatit VPOC1064 MD PH^®^) was purchased from LANXESS (Köln, Germany).

Dimethylsulfoxid-d_6_ (DMSO-d_6_) (≥99%) was purchased from Merck (Darmstadt, Germany).

### 2.2. Raw Material

Brewer’s spent grain (BSG) was provided by a local brewing company (ECM, Empresa de Cervejas da Madeira). The samples were freeze-dried immediately after delivery to our laboratory (NatLab—CQM). Dry BSG was stored at −20 °C until use. These samples were used throughout the work with the simple designation of “BSG”. A sample of fresh BSG was used for moisture and ash content determinations.

### 2.3. Physicochemical Characterization

The moisture content determination was carried out on a KERN DBS 60-3 moisture balance running a semi-automatic program, which heats the sample at 120 °C until the moisture content is stable for 30 s. Ash content (%) was determined after incineration of BSG samples in a muffle furnace at (500 °C for 24 h). Particle size distribution was studied by passing three freeze-dried BSG portions though a set of sieves with decreasing mesh size (1.0, 0.5, 0.25 and 0.125 mm).

### 2.4. Alkaline Hydrolysis—Basic Procedure

**Figure 1:**
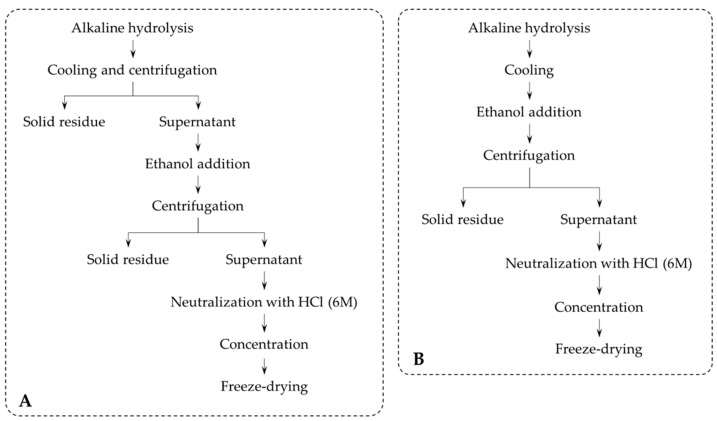
Proceeding to the alkaline hydrolysis reaction. (**A**) classic procedure; (**B**) simplified methodology.

Alkaline hydrolysis was performed according to the procedure outlined in Figure 1A and described in literature. Reactions were performed in Ace pressure tubes using BSG and NaOH solution in a solid:liquid ratio of 1:20 (*w*/*v*). Experimental conditions were optimized and set as 120 °C for 1.5 h with 20 mL of NaOH (2%) for 1 g of BSG. Optimization was performed by individual variation of each of the extraction parameters.

The tubes were placed into an oil bath and heated. After alkaline hydrolysis reaction, the mixture was cooled to room temperature, and the solid residue, containing mainly cellulose and lignin, was separated by centrifugation. Precipitation of the hemicellulose fraction was triggered by addition of ethanol to a final concentration of 30% (*v*/*v*), and its separation was performed by centrifuging. The supernatant, which contained the ferulic acid, was neutralized with HCl (6M) and concentrated on a rotary evaporator. The aqueous phase was freeze-dried and stored at −20 °C until analysis.

### 2.5. Pretreatment by Solid–Liquid Extraction with Acetone

A portion of BSG was extracted with acetone (60%) in a solid/liquid ratio of 50 mL/g in an ultrasound bath for 1 h. After solid–liquid extraction, the mixture was allowed to cool to room temperature, and the solid residue was separated from the supernatant by centrifugation followed by filtration. The solid residue was washed with distilled water and freeze-dried. Alkaline hydrolysis was carried out in Ace tubes at 120 °C for 1.5 h with 20 mL of NaOH (2%) for 1 g of BSG. For comparison, a portion of BSG not subjected to pretreatment was extracted by alkaline hydrolysis in the same conditions.

### 2.6. Extraction by Alkaline Hydrolysis in Autoclave

An alkaline hydrolysis assay was carried under the same conditions (120 °C for 1.5 h with 20 mL of NaOH (2%) for 1 g of BSG) in autoclave. The subsequent procedure was similar to that described for Ace tubes. The resultant extracts were stored at −20 °C until analysis.

### 2.7. Simplification of the Procedure

A modification of the process described in the literature was introduced in order to diminish the number of separation steps and thus improve the yield in the desired product; after alkaline hydrolysis, the mixture was cooled, and ethanol was added (Figure 1B). Precipitated hemicellulose was separated by centrifugation together with lignin and cellulose fraction. The supernatant was neutralized, concentrated, and the aqueous phase was freeze-dried and stored at −20 °C until analysis.

### 2.8. Analysis of Extracts

The extracts obtained after the procedures described in the previous sections were analyzed in terms of their total soluble solids (TSS), total phenolic content (TPC), and quantification of ferulic acid by HPLC-diode-array detector (DAD).

#### 2.8.1. Total Soluble Solids (TSS) Determination

For TSS determinations [25], extracts were resuspended in water (10 mg/mL) and filtered through membrane filters (0.45 μm). Using an ATAGO RX-1000 refractometer, the TSS was measured based on a calibration curve of sucrose (5–50 mg/L). The results are expressed in milligrams of sucrose equivalent (SE) per 100 g of dry BSG.

#### 2.8.2. Total Phenolic Content (TPC) Determination

TPC was determined by Folin–Ciocalteu method [26]; fifty microliters of the sample in methanol (5 mg/mL) was mixed with 1.25 mL of Folin–Ciocalteu solution (1:10) and 1 mL of Na_2_CO_3_ (7.5%). The absorbance at 765 nm was measured after 30 min, and results were expressed as milligrams of gallic acid equivalent (GAE) per 100 g of BSG (dry weight).

#### 2.8.3. Quantification of Ferulic Acid

Ferulic acid concentrations were determined by high performance liquid chromatography (HPLC) using a UV detector (at 320 nm) and a Phenomenex Gemini C18 (5 μm, 250 × 0.3 mm i.d.) column. The HPLC analysis, adapted from Gouveia and Castilho (2011) [26], was performed on a Dionex ultimate 3000 series instrument (Dionex, Sunnyvale, CA, USA) coupled to a binary pump, a diode-array detector (DAD), an autosampler and a column compartment. Samples (5 mg/mL) were prepared in the mobile phase and filtered through 0.45 μm membranes (Millipore, Burlington, MA, USA). Then, samples were injected into the equipment under the following conditions: column at 30 °C, acetonitrile/0.1% formic acid as mobile phase (isocratic elution, 75:25), a flow rate of 400 μL/min, and injection volume of 10 μL. Results are expressed as mg of FA per 100 g of BSG (dry weight).

Identification was performed comparing retention times with those obtained from commercial ferulic acid standard. Quantification was based on the UV signal response at 320 nm, and the resultant peak areas in the chromatograms were plotted against concentrations obtained from standard. Calibration curve (5–100 mg/L) was prepared by diluting the stock solutions (1000 mg/L in methanol) with the initial mobile phase. Quantification was carried out by plotting peak area versus concentration (*R*^2^ = 0.9994).

#### 2.8.4. Statistical Analysis

All samples were assayed in triplicate (*n* = 3), and results are given as means ± standard deviations. Differences between means were tested by ANOVA using SPSS Statistics 22 software.

### 2.9. Purification by Adsorption on a Synthetic Resin

Synthetic resin (Lewatit VPOC1064 MD PH^®^) was used to promote adsorption of FA from the extract obtained after alkaline hydrolysis using the simplified method previously described (Figure 1B).

#### 2.9.1. Kinetic Studies with FA Standard

Before purification of alkaline hydrolysis extract, adsorption kinetic studies with FA standard were performed. During these studies, several conditions were tested in three different assays. In the first assay, a proportional variation of FA concentration and the amount of adsorbent were carried up within three different tests. A second assay was achieved in two tests, where different initial concentrations of FA were studied. Finally, in the third assay, the effect of temperature in the adsorption was evaluated. Two tests were performed at room temperature (22–25 °C) and 6 °C (controlled ice bath), respectively. Table 1 resumes the conditions of the different assays.

A supernatant sample was collected every 2 min in the first 10 min, every 10 min during the next 90 min, and every 20 min until 180 min of adsorption. After filtration of supernatants, FA concentration was determined. Adsorption percentage, the amount of FA adsorbed per gram of resin, and *C*/*C*_0_ were used to evaluate the adsorption process.

The equation below was applied to the experimental data, and the parameters *α*, *β*, *γ*, *δ* and *θ* were determined using the Solver Microsoft Excel add-in program.

CC0=α+β·e−tγ+δ·e−tθ

where *C*_0_ is the initial concentration of the absorbate (g/L), and *C* is the concentration at the time *t* (g/L).

#### 2.9.2. Purification of an Alkaline Hydrolysis Extract

**Figure 2:**
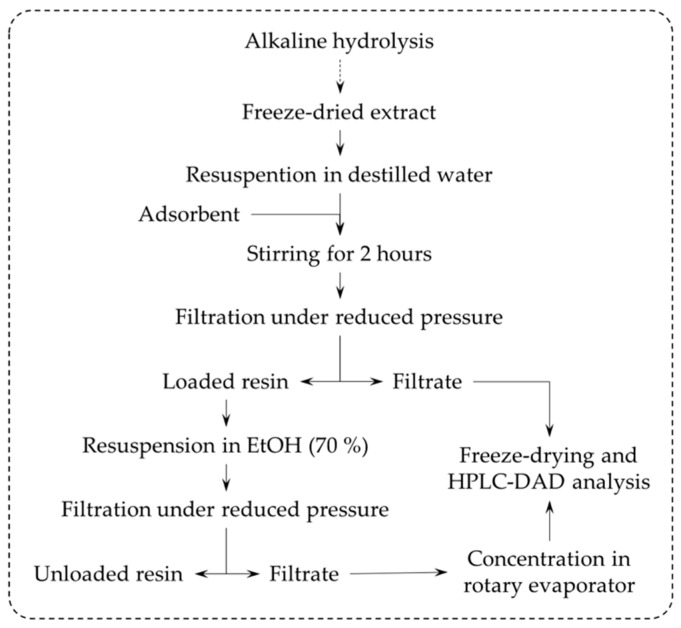
Procedure for the partial purification of FA by adsorption on a synthetic resin.

As outlined in Figure 2, a portion of 2 g of alkaline hydrolysis freeze dried extract was dissolved in 40 mL of water at 40 °C with vigorous stirring. After cooling the mixture to room temperature, a portion of 10 g of pre-activated resin (according to the supplier, treatment with 6% HCl and 4% NaOH and washing with distilled water) was added to the flask. The mixture was magnetically stirred for 2 h and filtered under reduced pressure. The filtrate was used to determine the FA adsorption yield, and the loaded resin was further stirred with 50 mL of ethanol:water (70%) in order to promote desorption. A new filtration allowed us to separate the resin from the liquid phase containing the FA. The determination of desorbed FA was possible through analysis of the filtrate.

The extracts were analyzed in terms of FA quantification by HPLC-DAD according to the conditions mentioned before. ^1^H NMR was performed to verify the partial purification of the extract obtained by alkaline hydrolysis of BSG and treated with the Lewatit resin. For NMR analysis, 10 mg of each extract were dissolved in 1 mL of DMSO-d_6_ and transferred to 5 mm NMR tubes. ^1^H spectra were recorded on a Bruker UltraShield 400 Plus NMR (Bruker, Billerica, MA, USA) at 10,061 MHz and 400 MHz. Acquisition parameters for ^1^H were: size of fit 65 k; spectral width 4401 Hz; acquisition time 64 k; relaxation delay 1 s; number of scans 512.

## 3. Results and Discussion

### 3.1. Physicochemical Characterization of BSG

The moisture content of supplied BSG was 68.44 ± 0.93%, and the ash content was 4.18 ± 0.03%. Sifting of freeze-dried material revealed that BSG was provided as a fine powder with particle size between 1 and 0.25 mm (Table 2), and it was further used without any separation.

### 3.2. Optimization of Alkaline Hydrolysis Conditions

Table 3 summarizes the results for optimization of alkaline hydrolysis reaction in Ace pressure tubes. Different reaction temperatures (60, 80, 100, and 120 °C) were tested for 1 h with NaOH (2%). Reaction time (1 to 3 h) was tested at 100 °C using NaOH (2%). Optimization of alkali solution concentration was performed in reactions at 100 °C for 1 h. Optimal conditions were set as 120 °C for 1.5 h with 20 mL of NaOH (2%) for 1 g of BSG.

### 3.3. Pretreatment by Solid–Liquid Extraction with Acetone

The results compiled in Table 4 reveal that extraction with acetone is effective in removing free sugars from the matrix, resulting in a significant decrease in TSS for pretreated BSG compared with the untreated portion. FA concentration in the extracts obtained by alkaline hydrolysis of both untreated and pretreated BSG portions did not show a statistically significant difference. Results obtained in previous works [27] showed that solid–liquid extraction with 60% acetone is efficient in the extraction of free form compounds from BSG. The present data show that solid–liquid extraction is not efficient in the extraction of FA from lignocellulosic materials, since it is covalently bonded to their structure, but soluble solids such as mono and disaccharides are partially removed.

### 3.4. Extraction by Alkaline Hydrolysis in Autoclave

The characterization of the extracts (Table 5) showed that differences on TSS were not significant. However, there was a statistically significant increase of TPC and FA yield for those obtained by alkaline hydrolysis in autoclave compared to those obtained in Ace pressure tubes. This might have been due to a higher contact exchange between BSG and alkali solution in autoclave, resulting from the greater volume of the reaction vessels in which hydrolysis was performed. Taking into account the aspects mentioned above, alkaline hydrolysis of BSG in an autoclave might be an interesting process for a possible scale-up of the extraction process, since even everyday laboratory equipment is capable of processing large amounts of BSG.

### 3.5. Simplification of the Procedure

Table 6 shows the results for the simplification of the procedure after alkaline hydrolysis in comparison with the normal procedure (schematized in Figure 1). Both TSS and TPC showed a large increase (of about 30.9% for TSS and 122.97% for TPC). FA yield was increased in about 80% with the change of procedure, up to 476.99 ± 25.94 mg (FA)/100 g (BSG, dry weight). The increase was probably due to the solvent washing during the addition of the ethanol to the alkaline liquor, which resulted in reduction of losses associated with the process. Thus, in addition to reducing the experimental steps and the energetic resources required for extraction, simplification of the procedure also permits obtaining a greater amount of FA. TSS increase may be an indication that larger carbohydrates are degraded into smaller, soluble sugar molecules.

### 3.6. Purification by Adsorption on a Synthetic Resin

#### 3.6.1. Kinetic Studies with FA Standard

Three assays were performed to study the adsorption kinetics under different experimental conditions, according to Table 1.

After determination of FA concentration in the various supernatant samples, *C*/*C*_0_ was calculated for each time *t*, and parameters *α*, *β*, *γ*, *δ* and *θ* were determined.

**Figure 3:**
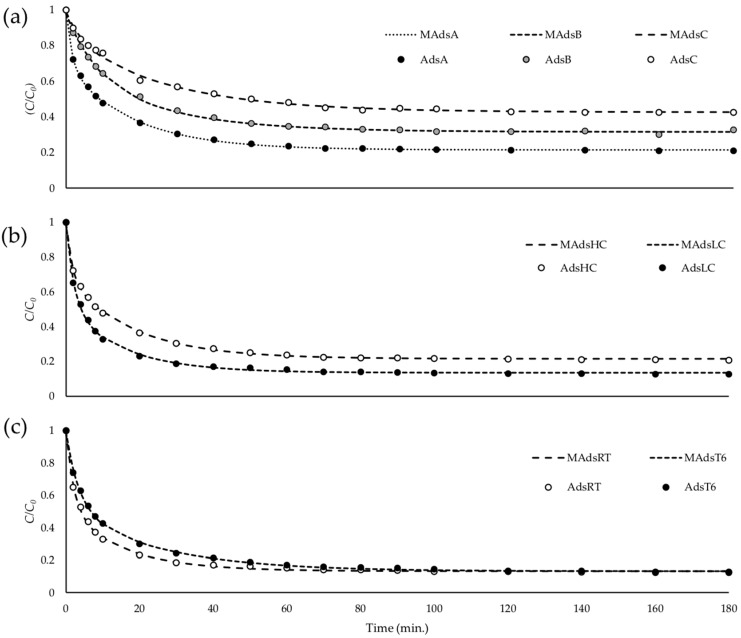
Adsorption kinetic isotherms for the assays performed to study the effect of (**a**) dispersion of FA and resin, (**b**) the initial concentration of FA, and (**c**) the temperature in the adsorption mixture.

Figure 3 resumes the evolution of *C*/*C*_0_ along the 180 min of adsorption in the different assays. Figure 3a is related to assay 1, where dispersion of both FA and resin increased in proportion within the tests *AdsA*, *AdsB*, and *AdsC*. Adsorption isotherms show that the variation in the concentration of adsorbed FA during the equilibrium was proportional to the dispersion of both adsorbate and adsorbent in the mixture. Assay 2 (Figure 3b) was performed to study the effect of the initial concentration of FA in the adsorption process. The FA concentration in test *AdsHC* was four times higher than that in test *AdsLC*. Because the amount of resin was similar in both tests, isotherms indicated a more efficient adsorption process for *AdsLC*. Regarding the study of the effect of temperature in the adsorption process, Figure 3c resumes the results obtained under room temperature (*AdsRT*) and 6 °C (*AdsT6*). Results showed that, at room temperature, the equilibrium was achieved faster than at 6 °C. In all tests, it was possible to establish that 100–120 min is enough to reach equilibrium.

Parameters for adsorption isotherms equations were determined and compiled in Table 7. Based on kinetic studies results, the best conditions for adsorption of FA standard may be an intermediate between the conditions of assays *AdsA*, *AdsLC*, and *AdsRT*. For real samples, where the goal is to purify FA from a variety of compounds of different chemical nature, the ideal conditions might be different, namely because of the competition for adsorption in the resin.

#### 3.6.2. Purification of an Alkaline Hydrolysis Extract

Adsorption of FA from the alkaline hydrolysis extract showed to be effective (90.83%), where around 4.6 milligrams of compound were adsorbed into the resin. The desorption was achieved by adding two portions of 25 mL of 70% ethanol followed by continuous stirring for 30 min and filtration. The filtrates were combined, concentrated, and the concentration of FA was determined by HPLC-DAD. Results showed that 68.70% of the adsorbed FA was desorbed under these conditions, indicating that about 1.4 milligrams of FA remained adsorbed.

**Figure 4:**
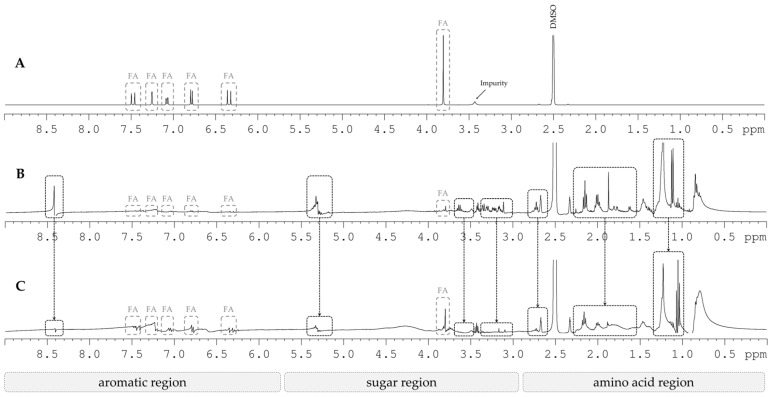
^1^H NMR spectra for (**A**) ferulic acid standard, (**B**) initial extract obtained by alkaline hydrolysis of BSG in autoclave with simplified post-extraction process and (**C**) final extract partially purified by adsorption/desorption in the Lewatit resin. Peaks corresponding to ferulic acid are marked in a grey box with the inscription “FA”. The black boxes indicate the regions where the differences were more significant. The arrows make the correspondence between initial and final extracts.

^1^H NMR spectra (Figure 4) suggests a partial purification of the extract obtained by alkaline hydrolysis of BSG in an autoclave and a simplified procedure. Figure 4A corresponds to FA standard spectrum. FA peaks were identified in both Figure 4B and Figure 4C for initial and partially purified extracts, respectively. Because FA concentration increased after purification, the peaks between 6.3 and 7.5 ppm and the peak at 3.8 ppm slightly increased in the final extract when compared to the original extract. On the contrary, peaks at 8.4 and 5.3 ppm as well as regions 3.5 to 3.7, 3.0 to 3.4, 2.6 to 2.8, 1.5 to 2.3, and 1.0 to 1.3 ppm decreased in the final extract compared with the initial extract. This suggests a decrease in the concentration of these compounds after partial purification with synthetic resin.

## 4. Conclusions

Because of its high availability and potential, several works are being developed around BSG. One of the interesting compounds present in the lignocellulosic structure of BSG is ferulic acid. Since FA is not extracted from the BSG matrix by conventional solid–liquid extraction methods, alternative techniques such as alkaline hydrolysis are often applied. The methods described in literature usually comprise a pretreatment step to remove compounds resulting from the brewing process. Alkaline hydrolysis is commonly followed by centrifugation, precipitation of hemicellulose fraction in the alkaline liquor, and neutralization. In the present work, the use of autoclave to perform the alkaline hydrolysis and a simplification of the post-extraction process were suggested and were shown to increase FA yield. The extraction in an autoclave resulted in an increase of FA extraction yield of around 38% when compared with the extraction in pressure tubes. An increase of about 84% in the extraction yield was achieved in a small scale extraction in pressure tubes when a simplification of the post-extraction process was applied. A partial purification by adsorption on a synthetic resin was also suggested, constituting a potential approach to obtain ferulic acid in a higher degree of purity.

During the studies, *p-*coumaric acid was co-extracted in molar proportion (*p-*CA:FA) between 1:4 and 1:8, depending on the extraction conditions.

## Figures and Tables

**Table 1:** Conditions of the adsorption mixtures during in adsorption kinetic studies. The volume of ferulic acid (FA) solution used in each teste was constant (50 mL).

Assay	Test	[FA] (g/L)	Resin wt. (g)	Temperature
1	*AdsA*	1	0.5	Room temp.
	*AdsB*	0.5	0.25	
	*AdsC*	0.25	0.125	
2	*AdsHC*	1	0.5	Room temp.
	*AdsLC*	0.25	0.5	
3	*AdsRT*	0.25	0.5	Room temp.
	*AdsT6*	0.25	0.5	6 °C

**Table 2:** Particle size distribution.

Particle Size (mm)	Percentage (%)		
>1	3.83	±	0.37
1–0.5	36.00	±	1.48
0.5–0.25	41.06	±	2.44
0.25–0.125	12.57	±	2.65
≤0.125	6.76	±	1.12

**Table 3:** Conditions and results obtained for optimization of the parameters for alkaline hydrolysis reaction in Ace pressure tubes. FA yield is expressed as mg of FA per 100 g of brewer’s spent grain (BSG).

Temp. (°C)	Time (Hours)	NaOH (%)	FA Yield (mg FA/100 g)		
60	1	2	46.95	±	9.72
80	1	2	81.52	±	17.89
100	1	2	214.18	±	30.43
120	1	2	234.98	±	8.03
100	1	2	214.67	±	4.70
100	1.5	2	223.97	±	6.82
100	2	2	223.05	±	1.47
100	2.5	2	202.62	±	19.65
100	3	2	223.09	±	17.85
100	1	0.5	174.76	±	23.64
100	1	1	199.63	±	11.21
100	1	1.5	214.53	±	23.26
100	1	2	204.44	±	9.64
100	1	2.5	200.10	±	22.07

**Table 4:** Comparison of the results obtained for alkaline hydrolysis of not pretreated and treated BSG (1 h). Total soluble solids (TSS) is expressed as mg of sucrose equivalent per 100 g of BSG, total phenolic content (TPC) is expressed as gram of gallic acid equivalent per 100 g of BSG, and FA yield is expressed as mg of FA per 100 g of BSG.

	Not Pretreated			Pretreated		
**TSS** mg SE/100 g	94.50	±	8.39 ^a^	69.39	±	3.28 ^b^
**TPC** g GAE/100 g	1010.44	±	1.58 ^a^	1323.24	±	143.30 ^b^
**FA yield** mg FA/100 g	259.21	±	35.95 ^a^	270.32	±	65.86 ^a^

^a^ indicates not significant differences and ^b^ indicates significant differences. SE: sucrose equivalent; GAE: gallic acid equivalent.

**Table 5:** Comparison of the results obtained for alkaline hydrolysis on Ace pressure tubes and in an autoclave (1.5 h). TSS is expressed as mg of sucrose equivalent per 100 g of BSG, TPC is expressed as gram of gallic acid equivalent per 100 g of BSG, and FA yield is expressed as mg of FA per 100 g of BSG.

	Ace Pressure Tubes			Autoclave		
**TSS** mg SE/100 g	81.45	±	2.59 ^a^	82.44	±	9.17 ^a^
**TPC** g GAE/100 g	1194.20	±	21.34 ^a^	1439.73	±	102.02 ^b^
**FA yield** mg FA/100 g	203.41	±	5.37 ^a^	280.61	±	5.77 ^b^

^a^ indicates not significant differences and ^b^ indicates significant differences.

**Table 6:** Comparison of the results obtained for normal and simplified procedures applied after alkaline hydrolysis. TSS is expressed as mg of sucrose equivalent per 100 g of BSG, TPC is expressed as gram of gallic acid equivalent per 100 g of BSG, and FA yield is expressed as mg of FA per 100 g of BSG.

	Normal Procedure			Simplified Procedure		
**TSS** mg SE/100 g	94.50	±	8.39 ^a^	123.70	±	1.47 ^b^
**TPC** g GAE/100 g	1483.72	±	90.03 ^a^	3342.86	±	71.21 ^b^
**FA yield** mg FA/100 g	259.21	±	35.95 ^a^	476.99	±	25.94 ^b^

^a^ indicates not significant differences and ^b^ indicates significant differences.

**Table 7:** Determination of parameter estimates and sum of squared differences (SSD) by MS Solver for adjustment of adsorption kinetic curves.

Assay	Test	Parameter					SSD
		*α*	*β*	*γ*	*δ*	*θ*	
**1**	*AdsA*	0.2144	0.4721	18.1123	0.3122	1.6490	0.0006
	*AdsB*	0.3169	0.3620	24.1647	0.3031	7.9226	0.0014
	*AdsC*	0.4262	0.4271	27.1620	0.1391	4.1121	0.0022
**2**	*AdsHC*	0.2144	0.4721	18.1123	0.3122	1.6490	0.0006
	*AdsLC*	0.1343	0.3759	15.7397	0.4861	2.2909	0.0014
**3**	*AdsRT*	0.1343	0.3759	15.7401	0.4861	2.2910	0.0014
	*AdsT6*	0.1335	0.3992	24.5310	0.4604	3.4751	0.0013
